# Two-Week Rehabilitation with Auditory Biofeedback Prosthesis Reduces Whole Body Angular Momentum Range during Walking in Stroke Patients with Hemiplegia: A Randomized Controlled Trial

**DOI:** 10.3390/brainsci11111461

**Published:** 2021-11-03

**Authors:** Dai Owaki, Yusuke Sekiguchi, Keita Honda, Shin-Ichi Izumi

**Affiliations:** 1 Department of Robotics, Graduate School of Engineering, Tohoku University, Miyagi 980-8579, Japan; 2 Department of Physical Medicine and Rehabilitation, Graduate School of Medicine, Tohoku University, Miyagi 980-8575, Japan; yusuke.sekiguchi.b2@tohoku.ac.jp (Y.S.); keita.honda.0528@gmail.com (K.H.); izumis@med.tohoku.ac.jp (S.-I.I.); 3 Graduate School of Biomedical Engineering, Tohoku University, Miyagi 980-8575, Japan

**Keywords:** auditory biofeedback, effect prediction, statistical modeling, stroke, two-week rehabilitation effect, walking rehabilitation

## Abstract

Walking rehabilitation is challenging in stroke patients with sensory impairments. In this study, we examined the two-week effect of an auditory biofeedback prosthesis, *Auditory Foot* (AF), on the change in the frontal whole body angular momentum (WBAM) range, before and after a two-week walking rehabilitation. We conducted a pilot randomized controlled trial (RCT). We employed statistical Bayesian modeling to understand the mechanism of the rehabilitation effect and predict the expected effect in new patients. The best-performing model indicated that the frontal WBAM range was reduced in the AF group by 12.9–28.7%. This suggests that the use of kinesthetic biofeedback in gait rehabilitation contributes to the suppression of frontal WBAM, resulting in an improved walking balance function in stroke patients.

## 1. Introduction

The main symptom of stroke due to brain damage is motor paralysis, e.g., gait disorder, which is the main cause of disability [1]. Patients with stroke require rehabilitation to regain functional capacity and to return to work [2]. Walking rehabilitation for patients with gait disorders directly improves the quality of life (QOL); thus, research on walking rehabilitation is of great social importance, potentially resulting in considerable benefit for patients with chronic stroke.

During rehabilitation, *kinesthesia*, that is, motion perception, that “how I am moving now”, which is generated through the interaction dynamics between motor and sensory systems, plays a crucial role in long-term motor learning as well as short-term motion generation. Thus, achieving kinesthesia is essential for the rehabilitation of physical impairments and disabilities. However, sensory impairments caused by neurological or physical disorders hamper kinesthesia, making walking rehabilitation difficult. Most studies have reported the effect of impaired plantar sensation on gait plasticity due to aging [3] or diseases, such as diabetes mellitus [4] or congenital insensitivity to pain with anhidrosis (CIPA) [5], and stroke [6,7,8].

Somatosensory impairment has been reported to affect motor recovery, gait, and balance rehabilitation in stroke patients [9]. Previous studies have proposed auditory feedback systems for walking rehabilitation; Miyake [10] proposed the Walk-Mate system that utilizes the “mutual entrainment” of the timing of footsteps of a subject and an agent modeled on a computer system and showed that patients’ as well as healthy subjects’ gait were restored to a stable and natural walking state. Schauer and Mauritz [11] verified the timing effect of auditory signals at touchdown during walking rehabilitation for stroke patients. Keasar and his group reported the short-term effects of gait rehabilitation in stroke patients using a visual and audio feedback system to increase the anterior ground reaction force [12,13]. However, no previous studies focused on transforming the spatiotemporal pattern of loading on a foot to auditory feedback signals.

For the walking rehabilitation of sensory impairments, we proposed an auditory biofeedback prosthesis [14], called *Auditory Foot* (AF), that transforms weak or deficient kinesthetic feedback into an alternative sensory modality. We focused on an auditory biofeedback from cutaneous plantar sensation for the following reasons: (i) plantar sensation, that is, the trajectory of the center of pressure (COP) on the plantar region and the magnitude of load, is an essential kinesthesia in walking [15,16]; (ii) in stroke patients with hemiparesis, the range of COP trajectories during walking is narrowed on the affected foot through the change of gait [17]; (iii) the time required for the cognitive resolution of auditory signals in the human brain (approximately 1 ms) is shorter than that required for the resolution of visual feedback signals (approximately 50–100 ms); and (iv) visual feedback systems, that is, a display showing visual feedback signals, constrains the posture of subjects, resulting in limited rehabilitation spaces and approaches. In our previous study [18] for 1-day short-term walking rehabilitation with AF in stroke patients, we found significant differences in the maximum hip extension angle and ankle plantar flexor movement on the affected side during the stance phase, between conditions with and without auditory feedback signals, suggesting that AF brought a short-term effect of improving the dynamical properties of gait in stroke patients. In this study, we attempt to verify the effect of a two-week walking rehabilitation on auditory biofeedback in stroke patients.

## 2. Materials and Methods

In this study, we tested the effect of a two-week walking rehabilitation on auditory biofeedback in stroke patients. To this end, we randomized 19 patients into an AF group with auditory feedback and a CT (control) group without feedback, and then performed gait rehabilitation for a two-week duration. To evaluate the two-week rehabilitation effect, we focused on whole body angular momentum (WBAM) in the frontal plane while walking and calculated the range of WBAM change during one gait cycle as a criterion of dynamic walking balance function. To understand the underlying mechanism of the confirmed rehabilitation effect and to predict the expected rehabilitation effect in new patients, we modeled the changes in the WBAM before and after rehabilitation using a Bayesian statistical model and estimated the parameters in the models based on the measured data. Finally, we constructed four statistical models, including individual differences in the effects, and compared the prediction accuracies of the models using the widely applicable information criteria (WAIC) [19,20,21,22,23].

### 2.1. Auditory Foot: Auditory Biofeedback Prosthesis

**Figure 1:**
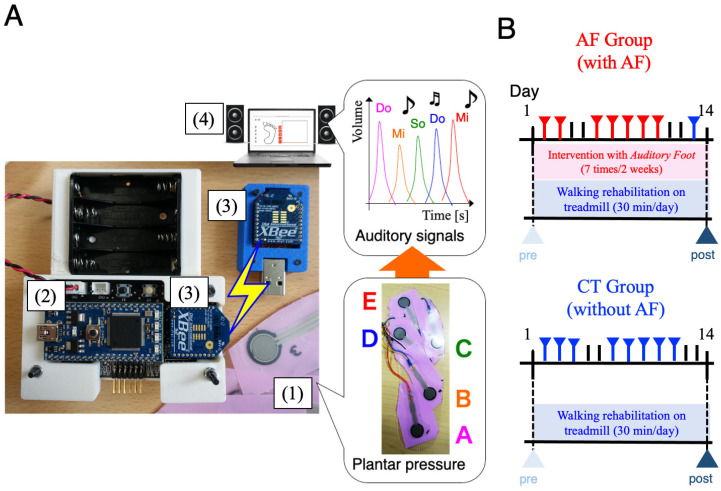
(**A**) Auditory biofeedback prosthesis, called *Auditory Foot*. Entire system of the sensor prosthesis which consists of four components: (1) pressure sensors (sensory input), (2) a microcomputer (sensor data processing), (3) wireless communication devices (data transportation), and (4) a PC (audio output). (**B**) Experimental design. The patients performed locomotion training (30 min/day) on a treadmill at a comfortable speed for two weeks. The subjects were randomized into two groups: AF group (training with AF) and CT group (training without AF as control). The subject of AF group performed seven rehabilitation sessions with AF during the two weeks. Red markers indicate AF intervention, blue markers indicate normal rehabilitation. The two black lines represent weekend, i.e., hospital was closed. As a buffer for a holiday during the two-week period, the 13th day contained normal walking training for the AF group.

We developed an auditory biofeedback prosthesis called AF for transforming sensory modalities during walking rehabilitation. AF transforms cutaneous plantar sensations to auditory feedback signals during walking. The entire system consists of four components (Figure 1A): (i) pressure sensors (input component, Interlink Electronics: FSR402), (ii) a microcomputer (data processing component), (iii) wireless communication devices (data transport component), and (iv) a PC (audio output component). The microcomputer (mbed NXP LPC1768) converted analog data from pressure sensors to digital data and sent them to a wireless communication device (XBee, Digital International: ZB RF module) via serial communication devices. Using XBee, digital data from the microcomputer were transported to a laptop PC via wireless communication. In the laptop PC, processing software [24] computes digital data from the XBee device and transformed them to auditory and visual outputs with a speaker and PC monitor, respectively. In processing software, we designed a transformation protocol from plantar sensation to auditory signal outputs as follows: the position of pressure sensors corresponded to a musical interval, e.g., Do, Mi, So, etc., and the magnitude of pressure sensor values corresponded to audio volumes. Thus, auditory signals corresponded to the spatiotemporal pattern of loading on a foot. The volume of the feedback sound was set to change analogically according to the magnitude of the pressure detected by the pressure sensor. The maximum volume was adjusted so that the patient could recognize the sound feedback during walking, using the plantar pressure of a normal healthy subject as a reference.

### 2.2. Participants and Protocol

From September 2015 to August 2019, we recruited subjects from the Department of Physical Medicine and Rehabilitation, Tohoku University Hospital, in Sendai, Japan. The inclusion criteria for the subjects included first-time stroke (caused by either an ischemic or hemorrhagic supratentorial lesion) and the ability to walk at least 7 min without using an assistive device. Exclusion criteria for both patients with hemiparesis and controls included the presence of brainstem or cerebellar lesions, a higher brain dysfunction (which would skew the measurements), and orthopedic problems. In patients, hemiparesis severity, the ability to perform movements outside the extensor and flexor, and synergy patterns were assessed using the Brunnstrom stages of recovery [25]. These tests were performed by an experienced physical therapist (Y.S.) while applying standardized protocols. This study was conducted in accordance with the tenets of the Declaration of Helsinki. All participants provided written informed consent before data collection, and study approval was granted by the institutional review board. The clinical trial ID was UMIN000018097 (https://upload.umin.ac.jp/cgi-open-bin/ctr_e/ctr_view.cgi?recptno=R000020945 accessed on 2 November 2021). Our research also follows the CONSORT guidelines (see the supplementary material: CONSORT checklist). For 1:1 randomization of 2 groups, participants were block randomized according to level of paresis and impaired sensory using Microsoft Excel (Microsoft, WA, USA) by an independent research assistant.

The patients performed locomotion training (30 min/day) on a treadmill at comfortable speed that they could maintain for 30 min for two weeks. Physical assistance was provided only as needed by a physical therapist for limb advancement, propulsion, and maintaining upright posture to prevent loss of balance. Patients used a handrail hold for balance and wore their habitual orthotic devices during every session. The symptoms of cardiorespiratory insufficiency, worsening neurologic impairments, or orthopedic injury were monitored by a physical therapist. If patients requested rest, they rested for approximately 1 to 2 min, once or twice a session. For all patients, physiotherapy continued with 1 physiotherapy and occupational therapy sessions daily, according to individual needs. The subjects were randomized into two groups: AF group (training with AF) and CT group (training without AF as control). The subjects of the AF group performed seven rehabilitation sessions with AF during the two weeks (Figure 1B top). The AF interventions in the AF group were performed on weekdays during the two-week hospitalization because rehabilitation could only be performed on hospital working days. On the first day of the hospitalization, initial gait assessment was performed. From the second day, gait rehabilitation using the AF was performed every weekday during the hospitalization. The final gait assessment was performed on the 14th day after 7 consecutive hospital working days of AF intervention, excluding Saturdays, Sundays, and holidays. As a buffer for a holiday during the two-week period, the 13th day contained normal walking training for the AF group. In a previous study, we showed a short-term effect of auditory biofeedback from two sensors, one at the heel and the other at the metatarsal, to improve walking kinematics and kinetic performance in stroke patients [18]. In the present study, we have used the same auditory feedback, wearing orthosis if patients used it. The subjects of the CT group performed similar sessions without AF during the two-week period (Figure 1B bottom). It was difficult for physical therapists and patients without auditory deficits to be blinded to the AF group; however, a blinded engineer analyzed gait parameters. This study was a single-blind randomized clinical trial comparing WBAM outcomes between treadmill training with and without AF.

### 2.3. Gait Analysis

For gait evaluation before and after the training, the subjects were asked to walk 7 min over two to ten trials. The patients were instructed to walk at a self-selected comfortable pace without assistive devices. The results comprise more than five strides during the successful trials. In addition, using adhesive tape, 41 reflective markers were attached to 12 segments. For all measurements, the MAC 3D System (120 Hz; Motion Analysis Corporation, Santa Rosa, CA, USA) was used to measure the coordinates of each reflective marker. The ground reaction force data were obtained at a 1200 Hz sampling rate using four 90 × 60 cm force plates (Anima Corporation, Chofu, Tokyo, Japan). The three-dimensional coordinates and ground reaction force data were smoothed using a bidirectional fourth-order Butterworth low-pass filter with cut-off frequencies of 6 and 80 Hz. This study used a 12-segment model based on anthropometric data, in accordance with the work of Dumas [26], which consisted of the feet, shanks, thighs, pelvis, thorax, upper arms, and forearms. For each joint in the lower extremities, the kinematic data were calculated using a joint coordinate system [27]. All data were time normalized to 100% of the one gait cycle. The parameters were calculated using a customized software program created with MATLAB (MathWorks Inc., Natick, MA, USA).

To examine the effects of two-week rehabilitation, we focused on balance assessment in stroke patients, especially by using the whole body angular momentum (WBAM) [28]. WBAM significantly reflects whole body dynamics during walking and contributes toward maintaining upright postural stability. Using kinematic data, frontal WBAM was calculated as the sum of angular momentum of each segment about a center of mass (CoM) described as follows:

(1) $$L_{WBAM}\left(t\right)=\sum_{i=1}^{12}L_{i}\left(t\right)=\sum_{i=1}^{12}\left\{\left(r_{CM}^{i}\left(t\right)-r_{CM}\left(t\right)\right)\times m_{i}\left(v_{CM}^{i}\left(t\right)-v_{CM}\left(t\right)\right)+I_{i}\omega_{i}\left(t\right)\right\},$$

where the first and second terms on the right hand side indicate transfer and local angular momentum, respectively, of each of the 12-segment human model without head (Trunk, Pelvis, Paretic(P)-upperarm, P-forearm, P-thigh, P-shank, P-foot, Non-paretic(N)-upperarm, N-forearm, N-thigh, N-shank, N-foot) with the use of kinematic date above. $r_{CM}\left(t\right)$ and $v_{CM}\left(t\right)$ indicate CoM position and velocity at *t* [% gait cycle], respectively. $m_{i}$, $r_{CM}^{i}\left(t\right)$, and $v_{CM}^{i}\left(t\right)$ indicate the mass, position, and velocity of the *i*th segment. $I_{i}$ and $\omega_{i}\left(t\right)$ represent inertia moment and angular velocity of the *i*th segment, respectively. Positive and negative values of the WBAM indicated the direction toward non-paretic and paretic sides, respectively. WBAM was normalized by the body mass (kg), height (m), and walking speed (m/sec), as these parameters affect the range of WBAM [28]. The range of the frontal WBAM, that is, the difference between the maximum and minimum values of WBAM during one gait cycle, is a useful evaluation index for gait balance in stroke patients:

(2) $$\begin{array}{c} L_{WBAM_{r}}=L_{WBAM_{max}}-L_{WBAM_{min}}=\max\left(L_{WBAM}\left(t\right)\right)-\min\left(L_{WBAM}\left(t\right)\right) \end{array}$$

### 2.4. Statistical Modeling

In rehabilitation of stroke patients, it is essential to consider *individuality*, that is, the individual differences among patients, to properly evaluate the rehabilitation effect. To investigate two-week effect of auditory feedback during rehabilitation by explicitly considering the individuality on patients, we used Bayesian statistical analysis and modeling. Bayesian model is a probabilistic model; thus, it is a good mathematical tool to model *uncertainty* on data, e.g., individual difference on patients. Bayesian analysis can estimate a probabilistic distribution (model) that encodes an unknown observation target by using observed data and updating the distribution in the model. Furthermore, a hierarchical (or multi-layer) model with a hyperparameter, which is a parameter for parameter, has a high affinity with Bayesian analysis and is a powerful tool to analyze data including individual differences. Thus, it is reasonable to apply Bayesian statistical modeling in the evaluation of rehabilitation for stroke patients, where individual differences can have a significant impact on the effectiveness.

Here, we modeled the relationship of the WBAM range on the pre and post two-week walking rehabilitation using four models (see the details in the next paragraph). We specified the models in probabilistic programming language Stan [29]. Here, we used non-informative uniform priors for some parameters unless described explicitly. For the estimation, we used a numerical method, Markov Chain Monte Carlo (MCMC), and scripted the models in R statistical environment (v.4.1.1) [30], in which the Stan code was compiled and executed using the R package “rstan” [29]. The software performed sampling from the prior distributions using No-U-Turn Sampler (NUTS) [31]. We decided the sampling convergence by trace plots and quantitative value, that is, the Gelman–Rubin convergence statistic $\hat{R}$ [32], where $\hat{R}<1.10$.

$L_{WBAR_{r}}^{pre}$ and $L_{WBAR_{r}}^{post}$ represent the WBAM range on pre and post condition of two-week rehabilitation, respectively. We here hypothesized that the effects of the two-week rehabilitation training are modeled as a linear relationship, that is, $L_{WBAM_{r}}^{post}=\beta \;L_{WBAM_{r}}^{pre}$, where parameter β denotes the training effect. The WBAM range increases for β>1, whereas the range decreases for β<1. The rehabilitation effect on the AF group includes the treadmill training effect, thus $\beta =\beta_{CT}+\beta_{AF}$, whereas for the CT group, $\beta =\beta_{CT}$ only. We assumed the distribution of $L_{WBAR_{r}}^{post}$ follows a normal distribution, described by the Normal (μ,σ) function, where μ and σ indicate the mean and standard deviation (S.D.) in the distribution, respectively. Indexes *i* and *j* represent the numbers of trials and patients, respectively.

**model 1: Non-hierarchical model**

(3) $$\begin{array}{c} L_{WBAR_{r}}^{post}{}_{,i}∼\mathrm{Normal}\left(\left\{\beta_{CT}+\beta_{AF}\right\}L_{WBAR_{r}}^{post}{}_{,i},\sigma \right), \end{array}$$

where $\beta_{CT}$ and $\beta_{AF}$ represent the effect of normal treadmill training for control patients and the additional effect with AF for the AF group patients, respectively. σ represents the standard deviation (S.D.) in the posterior distribution.

**model 2: Hierarchical model for $\beta_{CT}$**

(4) $$\begin{array}{ccc} \;L_{WBAR_{r}}^{post}{}_{,i,j} & ∼ & \mathrm{Normal}(\left\{\beta_{CT}{}_{,j}+\beta_{AF}\right\}L_{WBAR_{r}}^{pre}{}_{,i,j},\sigma ), \end{array}$$

(5) $$\begin{array}{ccc} \beta_{CT}{}_{,j} & ∼ & \mathrm{Normal}(\mu_{CT},\sigma_{CT}), \end{array}$$

We assumed the distribution of $\beta_{CT}{}_{,j}$, describing the individual differences in treadmill effects, follows a normal distribution, where $\mu_{CT}$ and $\sigma_{CT}$ indicate the mean and standard deviation (S.D.) in the distribution, respectively.

**model 3: Hierarchical model for $\beta_{AF}$**

(6) $$\begin{array}{ccc} \;L_{WBAR_{r}}^{post}{}_{,i,j} & ∼ & \mathrm{Normal}(\left\{\beta_{CT}+\beta_{AF}{}_{,j}\right\}L_{WBAR_{r}}^{pre}{}_{,i,j},\sigma ), \end{array}$$

(7) $$\begin{array}{ccc} \beta_{AF}{}_{,j} & ∼ & \mathrm{Normal}(\mu_{AF},\sigma_{AF}), \end{array}$$

We assumed the distribution of $\beta_{AF}{}_{,j}$, describing the individual differences in AF effects, follows a normal distribution, where $\mu_{AF}$ and $\sigma_{AF}$ indicate the mean and standard deviation (S.D.) in the distribution, respectively.

**model 4: Hierarchical model for $\beta_{CT}$ and $\beta_{AF}$**

(8) $$\begin{array}{ccc} \;L_{WBAR_{r}}^{post}{}_{,i,j} & ∼ & \mathrm{Normal}(\left\{\beta_{CT}{}_{,j}+\beta_{AF}{}_{,j}\right\}L_{WBAR_{r}}^{pre}{}_{,i,j},\sigma ), \end{array}$$

(9) $$\begin{array}{ccc} \beta_{CT}{}_{,j} & ∼ & \mathrm{Normal}(\mu_{CT},\sigma_{CT}), \end{array}$$

(10) $$\begin{array}{ccc} \beta_{AF}{}_{,j} & ∼ & \mathrm{Normal}(\mu_{AF},\sigma_{AF}), \end{array}$$

where $\beta_{CT}$ and $\beta_{AF}$ follow a normal distribution similar to model 2 and 3.

We compared the predictive performance of the constructed models by using a mathematical index, namely, the WAIC [21,22,23]. We were interested in predicting an “expected” rehabilitation effect for a new patient, not including our data. Bayesian model–based prediction of rehabilitation effects on new patients is essential for selecting rehabilitation methods and maintaining patients’ motivation. From this point, we here construct a new distribution of the expected effect of a new patient by marginalizing the intermediate parameters, $\beta_{CT}{}_{,j},\beta_{AF}{}_{,j}$, assigned to each hierarchical model (models 2–4) [23]. In the above described models, the model that shows the smallest WAIC value is the most appropriate predictive model in terms of rehabilitation effect for a new patient. Finally, we can find the best applicable model that describes the effect of auditory biofeedback rehabilitation including individuality on patients.

## 3. Results

**Figure 2:**
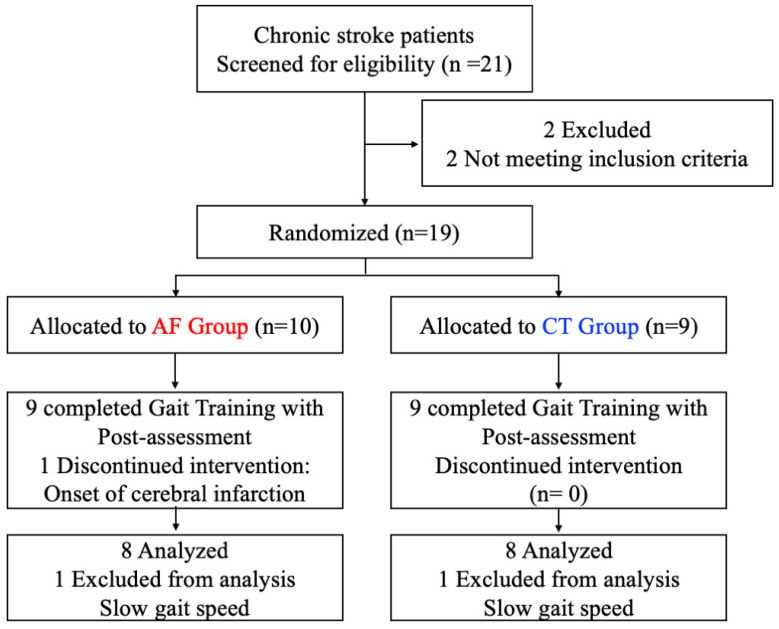
Nineteen patients after stroke participated in this study and eighteen patients completed the rehabilitation period. A patient dropped out for onset of cerebral infarction. Two additional patients were excluded from the following analysis because of their slow walking speed.

Nineteen patients after stroke participated in this study and eighteen patients completed the rehabilitation period. A patient dropped out for onset of cerebral infarction (Figure 2). Two additional patients were excluded from the following analysis because of their slow walking speed. Table 1 lists the demographic and clinical characteristics of the subjects. No significant between-group differences were found in the characteristics. The serious adverse events did not occur in the both groups.

### 3.1. Frontal WBAM during Gait Cycle

**Figure 3:**
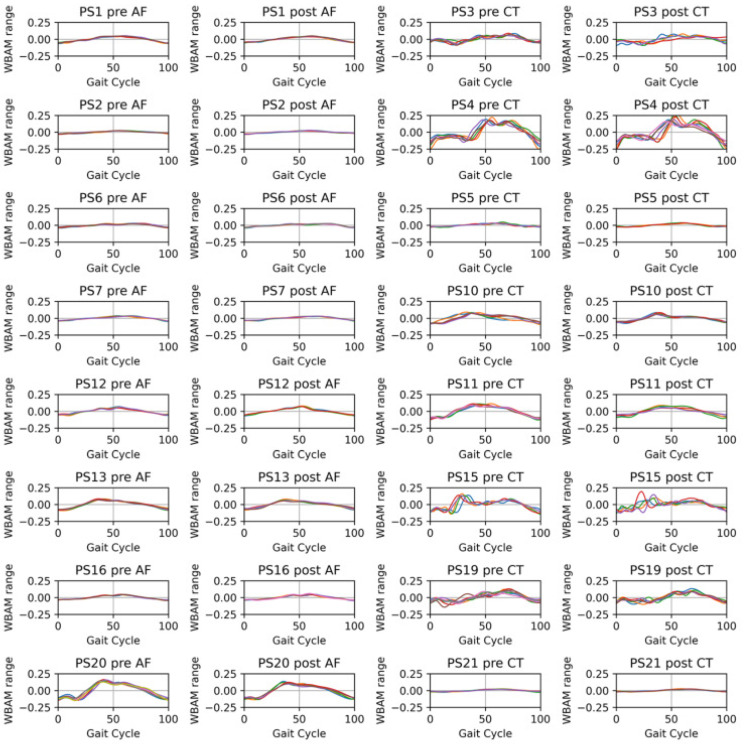
Frontal WBAM during one gait cycle for each patient on before (pre) and after (post) the training. From left to right, each panel indicates the precondition of the AF group, postcondition of the AF group, precondition of the CT group, and postcondition of the CT group. The positive direction of the vertical axis indicates the angular momentum around the CoM toward the non-paretic side. Gait cycle was set to 0% (100%) at the timing of the ground contact of the paretic foot. PS{} indicates the patient’s identification number.

Figure 3 shows the time series of frontal WBAM during one gait cycle ($L_{WBAM}\left(t\right)$, Equation (1)) for each patient on before (pre) and after (post) conditions of the rehabilitation. From left to right in Figure 3, each panel indicates the precondition of the AF group, postcondition of the AF group, precondition of the CT group, and postcondition of the CT group. The positive direction of the vertical axis indicates the angular momentum around the CoM toward the non-paretic side. Gait cycle was set to 0% (100%) at the timing of the ground contact of the paretic foot. PS{} indicates the patient’s identification number. In the precondition, the range of WBAM during one gait cycle varied depending on patients, indicating individual differences.

### 3.2. Frontal WBAM Range over Patients

**Figure 4:**
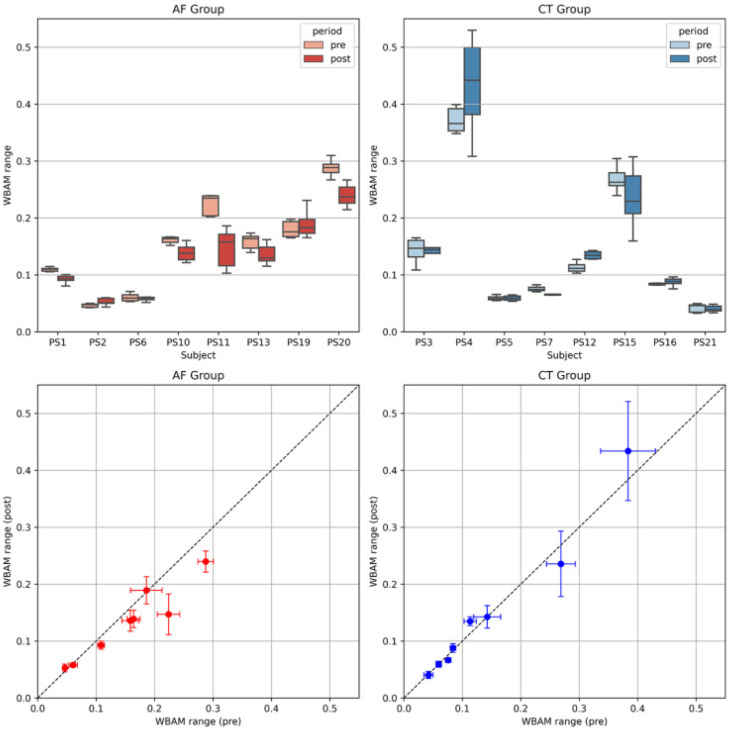
Frontal WBRA range on the pre and post conditions of two-week rehabilitation. The left and right panels show the AF and CT groups, respectively. The upper panels show boxplots of the changes in the pre and post conditions for each patient (including more than five gait cycles). The lower panels plot the mean and S.D. in the precondition on the horizontal axis and the postcondition on the vertical axis (pre–post plot of WBAM range). Each dot in the graphs represents the data for each patient. The black-dot lines indicate $L_{WBAM_{r}}^{post}=L_{WBAM_{r}}^{pre}$, which indicates that there is no change in the pre and post conditions.

Figure 4 shows the frontal WBRA range (difference between the maximum and minimum values during one gait cycle, $L_{WBAM_{r}}$ Equation (2)) on the pre and post conditions of rehabilitation. The left and right panels show the AF and CT groups, respectively. The upper panels show boxplots of the changes on the pre and post conditions for each patient (including more than five gait cycles). These results also indicate individual differences of the WBAM range in the precondition as “bias”, suggesting the difficulty of evaluation using normal statistical methods for stroke patients. The lower panels of Figure 4 plot the mean and S.D. in the precondition on the horizontal axis and the postcondition on the vertical axis (pre–post plot of the WBAM range). Each dot in the graphs represents the data for each patient. The black-dot lines indicate $L_{WBAM_{r}}^{post}=L_{WBAM_{r}}^{pre}$, which indicates that there is no change on the pre and post conditions of rehabilitation. These results indicate that the effects of the two week rehabilitation training are in a linear relationship, i.e., $L_{WBAM_{r}}^{post}=\beta \;L_{WBAM_{r}}^{pre}$, where the parameter β denotes the training effect. The rehabilitation effect on the AF group includes the treadmill training effect, thus $\beta =\beta_{CT}+\beta_{AF}$, whereas for the CT group, $\beta =\beta_{CT}$ only. In particular, the AF group is close to $\beta_{CT}+\beta_{AF}<1$ and the CT group is close to $\beta_{CT}\approx 1$. Based on these results, we constructed statistical models (Equations (3)–(8)), assuming individuality as hierarchical models, to predict the parameters $\beta_{AF},\beta_{CT}$, leading to the prediction of expected rehabilitation effects.

### 3.3. WAICs of Statistical Models

Table 2 shows the results of WAIC calculations for models 1 to 4: model 1 is a non-hierarchal model that does not take into account individual differences in $\beta_{CT}$ and $\beta_{AF}$, model 2 with individual differences in $\beta_{CT}{}_{,j}$, model 3 with individual differences in $\beta_{AF}{}_{,j}$, and model 4 also takes into account individual differences in both $\beta_{CT}{}_{,j}$ and $\beta_{AF}{}_{,j}$. The dWAIC shows the difference from the best-performed model (model 1), which has the lowest WAIC. In particular, the low prediction accuracy of models 2, 3, and 4, which take into account individual differences, suggests the fact that individual differences in the effects of treadmill and AF are significantly low, indicating that the individual “bias” in the precondition has a larger impact on the WBAM range after the rehabilitation training. In what follows, we discuss the prediction of rehabilitation effects using model 1.

### 3.4. Predicted Distribution of WBAM Range Via Bayesian Estimation

Table 3 summarizes the parameters of Bayesian predictive distribution for each model: means μ and prediction interval (95%) of the posterior distribution for $\beta_{CT}$ and $\beta_{AF}$. We hereafter focus on the prediction results of the best-performed model (model 1). The prediction interval for $\beta_{CT}$ (0.98931–1.10202) shows the effect of rehabilitation using the normal treadmill, performed by both groups, indicating that the WBAM range did not change before and after the rehabilitation. In contrast, in the AF group, the effect of rehabilitation was $\beta =\beta_{CT}+\beta_{AF}$, and thus, $\mu_{\beta_{CT}}+\mu_{\beta_{AF}}=0.83677$, and the 95% prediction interval was 0.7027 to 0.97265, indicating that the WBAM range was reduced due to the AF training in the two weeks.

**Figure 5:**
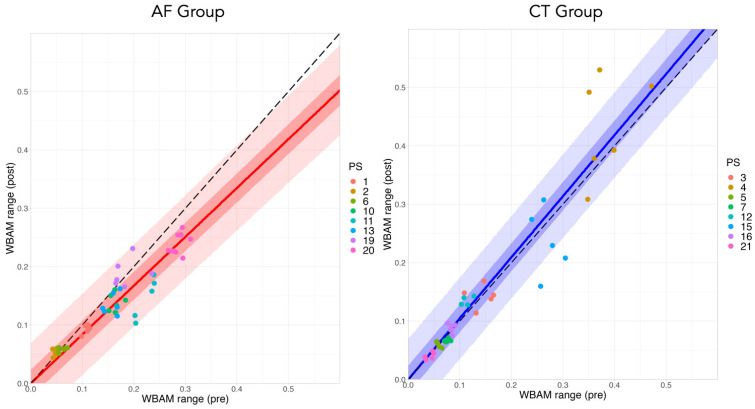
Bayesian predictive distribution on the pre–post plot of WBAM range. The mean (line), 50% prediction interval (dark color) and 95% prediction interval (light color) of the posterior distribution for model 1. The left and right panels show the AF and CT groups, respectively. Each point shows individual date of pre and post conditions for each trial. The different colors of the points indicate different patients’ data (see the legend), including more than five gait cycles. The black-dot lines indicate $L_{WBAM_{r}}^{post}=L_{WBAM_{r}}^{pre}$, which indicates that there is no change in the pre and post conditions.

Figure 5 shows the mean (line), 50% prediction interval (dark color), and 95% prediction interval (light color) of the posterior distribution in the pre–post plot of the WBAM range using the Bayesian model for model 1. The left and right panels show the AF and CT groups, respectively. Each point shows individual date of pre and post conditions for each trial. The black-dot lines indicate $L_{WBAM_{r}}^{post}=L_{WBAM_{r}}^{pre}$, which indicates that there is no change on the pre and post conditions. This figure shows that the Bayesian prediction intervals from model 1 adequately explain the data on pre and post rehabilitation for the patients. Furthermore, this figure for the AF group also indicates β<1, showing that the WBAM range was reduced by the AF rehabilitation.

## 4. Discussion

In this study, we examined the two-week effects of auditory biofeedback on gait rehabilitation in stroke patients. During a two-week gait rehabilitation training, we used *Auditory Foot* (AF) [14] which transforms plantar pressure sensation into auditory information, and analyzed the effects of the rehabilitation on the frontal WBAM range during walking. In general, “biofeedback” is defined as a method of feeding back information that is difficult to perceive by oneself, such as heartbeat and electroencephalogram (EEG), as other sensory information, e.g., sound, light. For patients with sensory impairment, it is difficult to perceive plantar pressure sensation by themselves. From this viewpoint, we call the AF an auditory biofeedback prosthesis. The posterior predictive distribution of the effect with a Bayesian statistical model showed that the WBAM range was reduced in the AF group, in the range of 12.9–28.7% (95% prediction interval, mean 20.9%), compared with the control (CT) group. Furthermore, comparison with hierarchical models, including individual differences for the parameters of the rehabilitation effects, showed that the model that predicted the posterior predictive distribution most accurately was the model that did not include individual differences, suggesting that the AF and treadmill training had little effect on individual differences in terms of the gait performance, that is, the WBAM range. In other words, individual differences in gait performance on post-rehabilitation do not significantly change during the rehabilitation process, but are largely influenced by the condition of each individual on pre-rehabilitation.

We employed WBAM as an evaluation criterion for two-week rehabilitation effects. Human upright bipedal walking is generated from whole body dynamic motion, suggesting that the coordination of movements between body segments significantly contributes toward maintaining dynamic walking stability. Gait asymmetry due to stroke, especially hemiplegia, has a significant impact on dynamic balance during walking, resulting in a 73% incidence rate of falls in post-stroke patients [34]; hence, balance assessment plays a crucial role in the diagnosis of stroke based on gait characteristics. Recent control methods in humanoid robots, for example, [35,36,37], use regulation of angular momentum in the frontal and sagittal plane for stabilization of the upright posture. Studies conducted on humans have also suggested that WBAM is strongly regulated by the central nervous system (CNS) [28,38,39,40] and is a “redaction” variable effectively representing whole body dynamics during human walking [41,42], and that angular momentum can be used as an effective balance assessment tool during steady-state hemiparetic walking [43,44].

Furthermore, recent studies have examined the effects of powered prosthesis or visual biofeedback on WBAM during walking in patients. A study that examined changes in WBAM in patients with a prosthetic powered lower-limb [45] reported that the powered prostheses could increase ankle power in the patients, whereas they could not bring the improvement for WBAM to the level of healthy subjects. A verification using visual feedback [46] also reported that WBAM became rather large, even when asymmetrical gait in post-stroke patients was suppressed by the biofeedback. In contrast to the visual feedback, which is a kinematic biofeedback, the plantar pressure sensation used in our auditory prosthesis is a somatosensory feedback, that is, kinestesia, fully reflecting the dynamics on patients’ walking. Our results suggest that the use of kinesthesia-related biofeedback in gait rehabilitation would contribute to the suppression of the WBAM, resulting in the improvement of the balance function in stroke patients.

Data-driven evaluation of rehabilitation effects is useful to understand the mechanisms behind the rehabilitation performed and, at the same time, to predict what kind of rehabilitation effects can bring in new patients. From this point of view, Bayesian statistical modeling is a highly effective approach to understand and predict the mechanisms behind complex phenomena by fitting data to a probability model. In addition, hierarchical Bayesian models can include individual differences in the hyperparameters, making it possible to predict the rehabilitation effect on a new patient with unknown gait characteristics. Recent studies examining the effects of the treadmill training have reported improvements in walking speed, endurance [47], walking distance [48], pelvic motion, and asymmetry of center of pressure (CoP) displacement [49]. Furthermore, many studies have discussed the limitations of the existing statistical methods [48,49]; these limitations can be attributed to the different recovery patterns of individual patients, that is, the influence of individual differences on pre-rehabilitation characteristics, in the effects of treadmill training, or in recovery effect from other rehabilitation training. In contrast, statistical modeling using the hierarchical Bayesian model that we adopted here is a novel attempt in that it can parameterize the pre-rehabilitation characteristics, the effect of treadmill training, and the effect of AF, and construct a model that assumes individual differences in each of them, leading to overcoming the limitations. The comparison of the prediction distributions using WAICs (Table 2) showed that the non-hierarchical model (model 1) had the highest prediction accuracy, suggesting that the individual differences rely on pre-rehabilitation characteristics and not on the treadmill training and AF training effects. Furthermore, the comparison between model 2 (considering individuality on $\beta_{CT}$, the treadmill training) and model 3 (considering individuality on $\beta_{AF}$, AF training) suggests that the effect of individual differences on the $\beta_{AF}$ would be relatively small, which may suggest a consistent effect of our AF biofeedback prosthesis during walking rehabilitation.

There are several limitations to the present study. First, we have not considered the carry-over effect of the treadmill and AF rehabilitation. Maintaining the training effect is an essential aspect of long-term rehabilitation for more effective rehabilitation. A report about a rhythmic motor learning task with visual and auditory signals [50] indicated that the visual feedback group became dependent on the feedback for their performance after the practice, whereas the auditory feedback group performed equally well with or without feedback after practice. This finding suggests that our auditory biofeedback prosthesis would eventually allow patients to be less reliant on auditory feedback for walking performance on the post-rehabilitation. Second, the effects of continuous AF rehabilitation over a longer period, such as several months or a year [51], should also be examined and discussed. In our experiment, we tested the effects of AF training for two weeks due to the limitation of the experimental protocol. In such a long-term rehabilitation, it is essential to maintain the patients’ motivation, which is necessary for accurately predicting the rehabilitation effect via the statistical Bayesian model. According to personal questioning of patients after rehabilitation, many patients did not feel annoyed and some even responded as if the auditory feedback was still present in their brain after rehabilitation. We consider this to be a sort of carry-over effect. Third, it is important to compare the long-term intervention effects of other sensory biofeedback, for example, visual, tactile feedback, or orthotics such as ankle foot orthosis (AFOs) [52,53], and to verify the effects of their combination. Fourth, our study lacked the diversity of patients. It may be difficult to generalize the results of this study to the broader stroke population because the sample size was relatively small. We calculated Cohen’s *d* value to examine the effect of sample size [54]. The variance of the two-week walking rehabilitation effect on the CT group ($\beta_{CT}$) and the AF group ($\beta_{CT}+\beta_{AF}$) was 0.16393718 and 0.19052502, respectively. Bayesian estimation is a method of estimating the posterior probability distribution of the parameters ($\beta_{CT},\beta_{AF}$) based on the measured data. Therefore, the *d*-value was also obtained as a probability distribution. The mean value of *d* on this distribution was d=1.173994(>1.0) for model 1, suggesting that the two-week walking rehabilitation of AF is effective despite the small sample size (16 patients). Fifth, our study was a single-blind trial. In a previous study [55], no evidence showed a difference in the estimated treatment effect between trials with and without blinded patients, healthcare providers, or outcome assessors. Therefore, a lack of blinding may not have significantly influenced the present results. Sixth, it is possible to build a Bayesian prediction model that takes into account parameters of various factors of clinical evaluation in medicine, such as SIAS sensory function scores and Berg Balance Score (BBS). The prediction from a statistical model with various parameters that should be considered for more accurate gait diagnosis and selection of intervention methods is expected to contribute to the establishment of a effective rehabilitation system. Therefore, in the future, we intend to establish a *Bayesian model-based rehabilitation* that provides optimal and predictable interventions for each individual patient.

## 5. Conclusions

Our statistical analysis using a Bayesian model showed that the WBAM range was reduced in the AF group by 12.9–28.7% (95% prediction interval, mean 20.9%) compared with the CT group. Furthermore, comparison with hierarchical models, including individual differences for the parameters of the rehabilitation effects, showed that the model that predicted the posterior predictive distribution most accurately was the model that did not include individual differences, suggesting that the AF and treadmill training had little effect on individual differences in terms of the gait performance. Furthermore, a comparison between model 2 (considering individuality on $\beta_{CT}$, the treadmill training) and model 3 (considering individuality on $\beta_{AF}$, AF training) suggests that the effect of individual differences on the $\beta_{AF}$ would be relatively small, which may suggest a consistent effect of our AF biofeedback prosthesis for the two-week walking rehabilitation.

The clinical significance of our results confirmed the two-week effect of auditory biofeedback on the frontal WBAM in stroke patients with hemiplegia and a sufficient post-stroke period, which was determined by the formation of an “abnormal” but stable gait pattern. These results are due to brain plasticity during rehabilitation. In this study, we showed that the reconstruction of kinesthesia resulted in the plastic stabilization of gait in patients with weakened sensory function due to stroke. These patients underwent a two-week walking rehabilitation program to compensate for plantar pressure sensation based on auditory biofeedback using our AF. This study provides valuable and important results for neurorehabilitation based on sensory compensation. However, the detailed mechanism of this rehabilitation effect based on brain plasticity requires further research.

## Figures and Tables

**Table 1:** Demographic and clinical characteristics of the subjects. No significant between-group differences were found in the characteristics.

Groups	AF Group	CT Group	*p*-Value
Age (years)	56.9 ± 10.8	56.6 ± 12.0	0.966
Height (cm)	169 ± 6.8	169 ± 8.6	0.945
Weight (kg)	68.7 ± 7.7	66.8 ± 13.8	0.732
Affected side			
(rt/lt)	3/5	5/3	0.317
Gender			
(male/female)	7/1	6/2	0.522
Stroke type			
(isochemic/hemorhagic)	2/6	5/3	0.131
Time from stroke onset (days)	1767 ± 2428	1937 ± 1547	0.870
SIAS*: total score	51 ± 8	52 ± 5	0.878
SIAS: sensory (lower limb)			
Touch	1.6 ± 0.5	2.4 ± 0.7	0.065
Position	2.4 ± 0.7	2.8 ± 0.5	0.161
Berg Balance Scale	48 ± 5	49 ± 5	0.721
6 min walk test (m)	326 ± 77	313 ± 115	0.797

SIAS*: Stroke Impairment Assessment Set [33].

**Table 2:** WAICs of the statistical models. The dWAIC shows the difference from the best-performed model (model 1), which has the lowest WAIC.

Model	Individuality	Rank	WAIC	dWAIC
model 1	no	1	−331.3894	0
model 2	$\beta_{CT}{}_{,j}$	2	−318.9331	12.4563
model 3	$\beta_{AF}{}_{,j}$	3	−266.1793	65.2101
model 4	$\beta_{CT}{}_{,j},\beta_{AF}{}_{,j}$	4	−190.9220	140.4674

**Table 3:** Parameters of Bayesian predictive distribution for each model. Means and prediction interval (95%) of the posterior distribution for $\beta_{CT}$ and $\beta_{AF}$.

Model	$\mu_{\beta_{\mathrm{CT}}}$	$\beta_{\mathrm{CT}}$: (95%)	$\mu_{\beta_{\mathrm{AF}}}$	$\beta_{\mathrm{AF}}$: (95%)
model 1	1.04607	0.98931–1.10202	−0.20930	(−0.28761) – (−0.12937)
model 2	1.01281	0.89266–1.13065	−0.16005	(−0.32005) – 0.00411
model 3	1.04554	0.99289–1.10028	−0.19355	(−0.32110) – (−0.05443)
model 4	1.01189	0.89506–1.12580	−0.15148	(−0.32501) – 0.03550

## Supplementary Materials

The following are available online at https://www.mdpi.com/2076-3425/11/11/1461/s1, Figure S1: Antibiogram of the CTX-M-15-producing Klebsiella pneumoniae responsible of the outbreak.

## Abbreviations

The following abbreviations are used in this manuscript:

WBAM	Whole Body Angular Momentum
AF	Auditory Foot
CT	Control
WAIC	Widely Applicable (Bayesian) Information Criterion
CoM	Center of Mass
CoP	Center of Pressure
SIAS	Stroke Impairment Assessment Set
